# The morphology of silver nanoparticles prepared by enzyme-induced reduction

**DOI:** 10.3762/bjnano.3.47

**Published:** 2012-05-18

**Authors:** Henrik Schneidewind, Thomas Schüler, Katharina K Strelau, Karina Weber, Dana Cialla, Marco Diegel, Roland Mattheis, Andreas Berger, Robert Möller, Jürgen Popp

**Affiliations:** 1 IPHT – Institute for Photonic Technology, Albert-Einstein-Strasse 9, D-07745 Jena, Germany; 2 Institute of Physical Chemistry, Friedrich Schiller University Jena, Helmholtzweg 4, D-07743 Jena, Germany; 3 Max Planck Institute of Microstructure Physics, Weinberg 2, D-06120 Halle, Germany

**Keywords:** EGNP, enzymatically grown silver nanoparticles, enzyme-induced deposition, nanoflower, SERS

## Abstract

Silver nanoparticles were synthesized by an enzyme-induced growth process on solid substrates. In order to customize the enzymatically grown nanoparticles (EGNP) for analytical applications in biomolecular research, a detailed study was carried out concerning the time evolution of the formation of the silver nanoparticles, their morphology, and their chemical composition. Therefore, silver-nanoparticle films of different densities were investigated by using scanning as well as transmission electron microscopy to examine their structure. Cross sections of silver nanoparticles, prepared for analysis by transmission electron microscopy were additionally studied by energy-dispersive X-ray spectroscopy in order to probe their chemical composition. The surface coverage of substrates with silver nanoparticles and the maximum particle height were determined by Rutherford backscattering spectroscopy. Variations in the silver-nanoparticle films depending on the conditions during synthesis were observed. After an initial growth state the silver nanoparticles exhibit the so-called desert-rose or nanoflower-like structure. This complex nanoparticle structure is in clear contrast to the auto-catalytically grown spherical particles, which maintain their overall geometrical appearance while increasing their diameter. It is shown, that the desert-rose-like silver nanoparticles consist of single-crystalline plates of pure silver. The surface-enhanced Raman spectroscopic (SERS) activity of the EGNP structures is promising due to the exceptionally rough surface structure of the silver nanoparticles. SERS measurements of the vitamin riboflavin incubated on the silver nanoparticles are shown as an exemplary application for quantitative analysis.

## Introduction

The application of metal nanoparticles in the field of bioanalytics extends the possibilities of biomolecular detection significantly and may satisfy the ever-growing interest in ultrasensitive detection methods for different applications [1]. Due to their interesting and unique properties, metal nanoparticles have the potential to meet the requirements for different biomolecular investigations based on optical, electrical, electrochemical, or gravimetric detection schemes [2–4]. Concerning their optical properties, metal nanoparticles are characterized by high extinction coefficients as well as large scattering cross sections, making them prospective candidates for optical approaches based on absorption or scattering processes [5]. Another simple and robust approach for the detection of biomolecules utilizing properties of metal nanoparticles is the measurement of the electrical conductivity. It was shown, that bioanalytical investigations can be carried out by measuring the electrical conductivity of accumulated metal nanoparticles in a gap between two electrodes [6–7]. A further promising application of metal nanoparticles concerning the synthesis of bioanalytically adaptive nanoparticles is the usage as reaction seeds for a specific reductive metal deposition process. The subsequent additional metal deposition leads to core–shell structures [8], which feature special properties that are well suited for the detection of biomolecules [9–11]. Even though the application of nanoparticles for the detection of biomolecules is already widespread, its full potential has not been exploited yet, since the performance of the metal nanoparticles as well as of core–shell particles strongly depends on their size, morphology, and composition, which have to be optimized for bioanalytical investigations.

A lot of effort has already been made by researchers to optimize and control the synthesis of nanoparticles in all types, concerning material, shape and size, and to characterize the nanoparticle properties [12–18]. One promising synthesis strategy for nanoparticles, particularly suited for bioanalytical problems, is the enzymatically catalysed formation of nanoparticles [19–21]. The characterization of such enzymatically grown silver nanoparticles by atomic force microscopy [6] revealed an unusual, but very characteristic morphology: the desert-rose-like structure. This rose-like structure appears soon after the reaction is started and can be observed even for large particle sizes above 0.5 µm. These special nanostructures, composed of different intertwined plates, show sharp and spiky features, thus comprising areas which are characterized by a strong electromagnetic-field enhancement due to the interaction of light with plasmonically active structures. Consequently, these nanostructures enable the realization of spectroscopic detection schemes, such as surface-enhanced Raman spectroscopy (SERS) [21]. The SERS activity of these enzymatically grown metallic nanostructures has been characterized with the help of a simple conductivity measurement [7].

Of course, there are a number of different techniques that can be used for the synthesis of desert-rose-like structures, among them galvanic displacement processes [22], wet chemical methods [23], particle-mediated aggregation [24], or electrodeposition on photolithographically prepatterned metal islands [25]. The structures composed of intersecting nanopetals or nanoplates are also called flower-shaped nanoparticles, nanoflowers, mesoflowers, or sea urchins in literature.

The aim of this work is to study the particle growth process with respect to particle shape, and the chemical composition with respect to possible contaminations. Therefore, the enzymatically grown silver nanoparticles were investigated by employing different microscopic techniques, namely scanning electron microscopy (SEM) and transmission electron microscopy (TEM), together with analytical methods, namely Rutherford backscattering spectrometry (RBS) and energy-dispersive X-ray spectroscopy (EDX). These studies were performed both on a single-particle level as well as on particle arrangements or films.

## Results and Discussion

In the following we report on investigations of the morphology and the composition of silver nanoparticles generated by means of an enzymatically induced redox reaction. The properties of the silver nanoparticles were characterized in dependence on the DNA concentration for binding of the enzyme and the reaction time in order to obtain samples with different densities and sizes of silver EGNPs.

**Figure 1:**
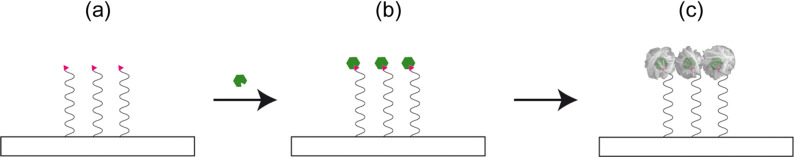
Schematic sketch displaying the growth mechanism of the silver EGNP: (a) the substrates are immobilized with single-strand DNA as linker molecules; (b) this is followed by binding of HRP at the target DNA; (c) finally the growth of the nanoflower-like silver nanoparticles is induced by the enzyme.

The principle of silver EGNP synthesis is shown in Figure 1. After the preliminary substrate cleaning and preparation, an amino-modified single-strand DNA was bound onto the substrate in order to act as seeds for the silver growth (a). In a second step, the enzyme horseradish peroxidase (HRP) is applied and bound to the DNA (b). Finally, the silver deposition is activated by an enzymatic reaction leading to the growth of silver particles at the enzyme (c).

In the enzymatic process an oxygen atom is split off from the hydrogen peroxide. This oxygen is bound to the heme group of HRP, thus changing the oxidation state of the iron atom inside the molecule. Within this process the electron donor releases electrons for the reduction of the metallic silver. This process takes place only in close vicinity to the enzyme.

### Size and distribution of the silver EGNP

**Figure 2:**
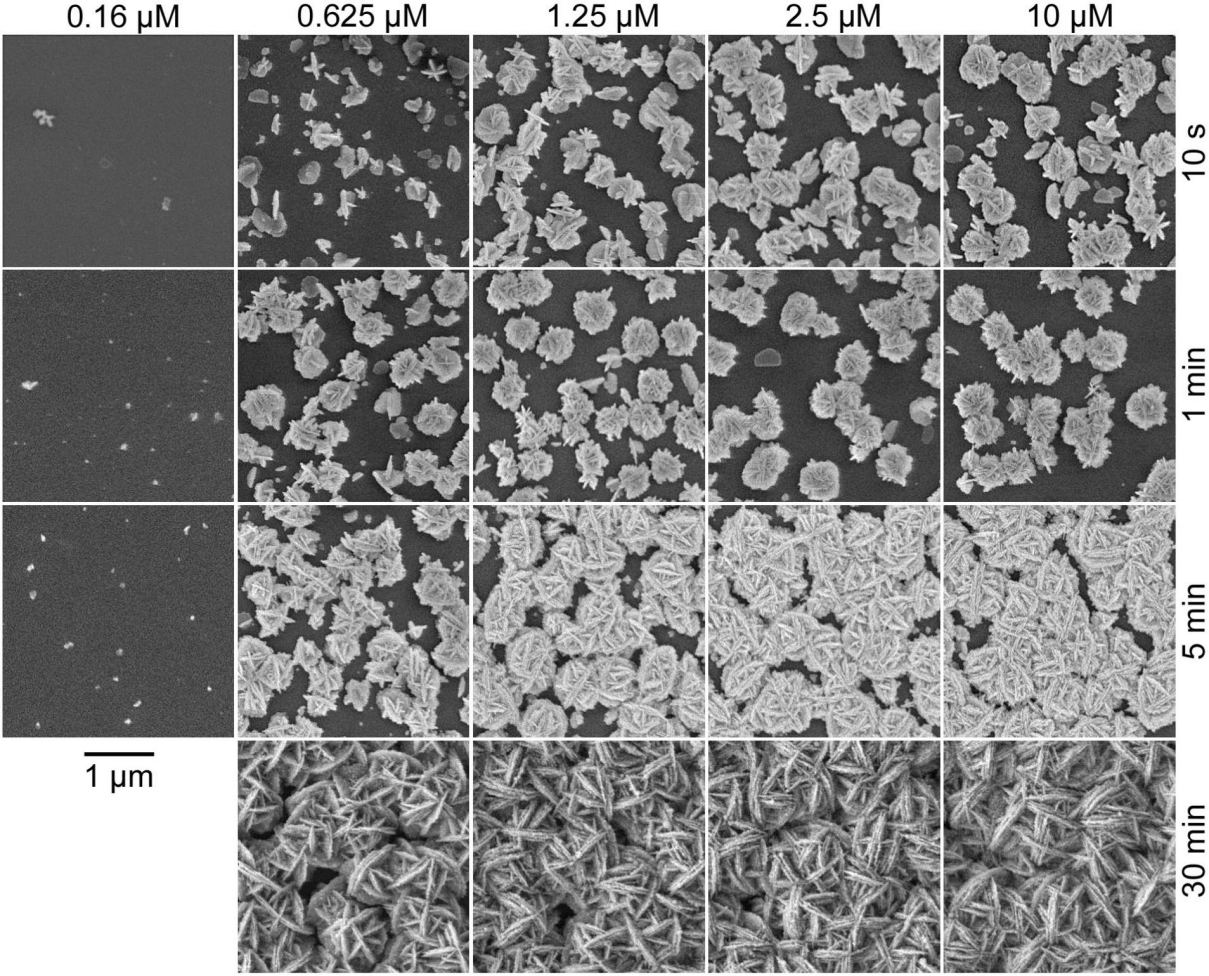
SEM images of silver EGNPs grown from different starting DNA concentrations of 0.16, 0.625, 1.25, 2.5, and 10 µM (as labelled above the columns). The reaction time was 10 s, 1 min, 5 min, or 30 min, respectively. The scale bar of 1 µm, as indicated, is valid for all parts of the figure. The images were taken at normal incidence.

In order to investigate the nucleation and growth of the silver EGNP we applied different starting concentrations of DNA between 0.16 µM and 10 µM. The DNA molecules act as seeds for the growth of the silver nanoparticles. In succession, HRP was added as a catalyst to initiate the growth of the silver nanoparticles. The concentration of HRP was 1:1000 for all samples with regard to an initial concentration of 1 mg/ml in a buffer solution. The growth of the silver nanoparticles was stopped after different reaction times ranging from 10 s to 30 min. Figure 2 shows SEM images of silver EGNP samples on glass substrates for different DNA concentrations as well as reaction times. Experiments with a starting concentration of the DNA below 0.5 µM resulted in only small EGNPs with a very low final density, even after rather long reaction times of several minutes. Increasing the DNA concentration above a medium transition range of approximately 0.5–1.0 µM led to dense arrays of EGNPs with the diameters of the single silver nanoparticles being several hundred nanometers up to one micrometer. Furthermore, above 1 µM DNA the size, shape, and distribution of the silver nanoparticles was almost independent of the starting DNA concentration. The silver nanoparticles show a remarkable morphology, known as nanoflowers or desert roses. They reveal interpenetrating plates standing on each other with sharp edges, resulting in an overall spherical shape with an enlarged surface.

Figure 2 shows that irrespective of the DNA concentration, two different appearances of silver nanoparticle arrays depending on the reaction time were observed. During the initial growth phase (typically below 1 min) single particles as well as some silver nanoparticles connected to each other can be found. Furthermore, silver nanoparticles consisting of intertwined silver plates are clearly observed. After a reaction time of one to five minutes, a rather dense array of silver nanoparticles, which are mostly connected to each other, was obtained. For DNA concentrations exceeding the aforementioned value of 0.5–1.0 µM, similar silver nanoparticles were generated with increasing density. The observation of no significant further growth after approximately 5 min is consistent with previous work, in which the maximum height of the silver nanoparticles was determined by AFM measurements [6]. Assuming that all unbound enzyme was rinsed away by a buffer solution before the synthesis began, we expect an inactivation of the enzyme activity due to geometrical shielding of the DNA–HRP complex with increasing thickness of the deposited silver. During the further growth, the silver nanoparticles act as seeds for an additional deposition, including nonspecific silver precipitation independent of the enzymatic reaction [26].

**Figure 3:**
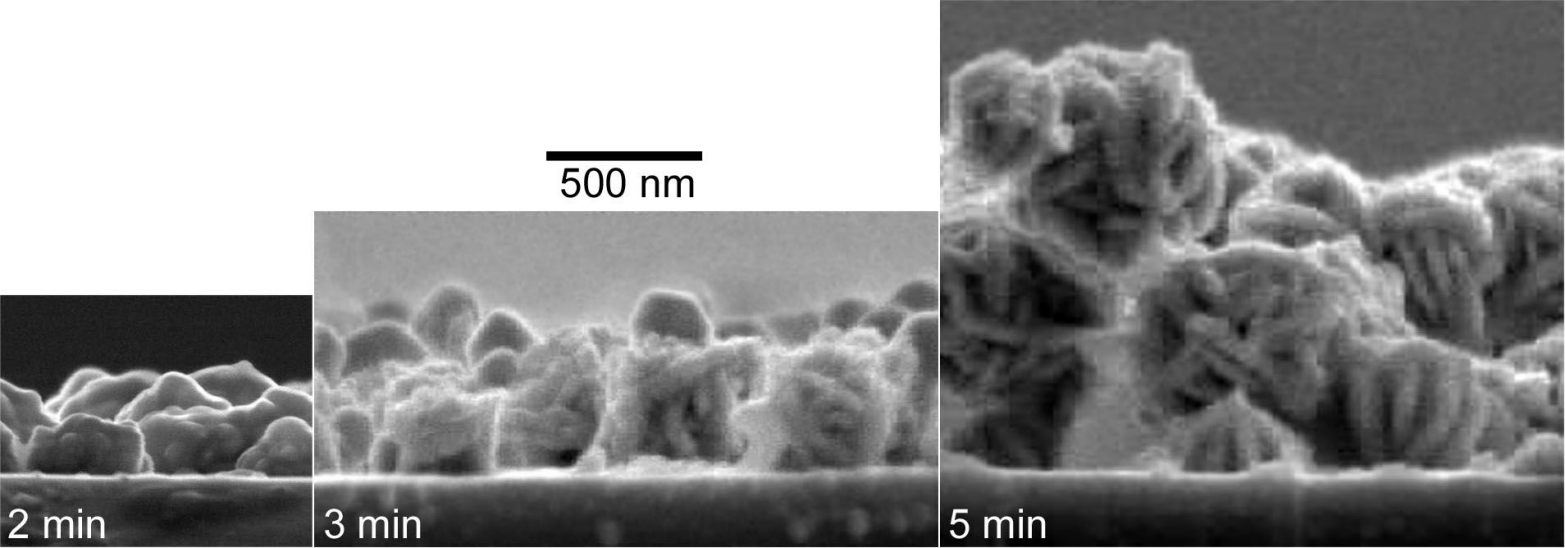
SEM cross-section images of silver EGNP grown from a starting DNA concentration of 25 µM. The reaction times were 2, 3, and 5 min, respectively. The scale bar of 500 nm as indicated is valid for all three images.

In order to obtain the size of the silver nanoparticles, cross-section SEM images were recorded. Figure 3 shows SEM images for a DNA concentration of 25 µM. It can be seen, that the height of the individual silver nanoparticles reaches approximately 100 nm, 250–400 nm, and up to 600 nm for reaction times of 2, 3, or 5 min, respectively. It is clearly observable, that the films are built from densely packed particles, which are partially stacked one above the other. The corresponding overall thickness of the dense film-like arrays composed of the silver nanoparticles peaks at 0.3, 0.7, or above 1 µm, respectively. Within the time scale considered in Figure 3, no saturation of the silver nanoparticle growth was reached.

The DNA concentration is one of the main factors influencing the resulting density of the silver nanoparticles. The DNA strands at the substrate surface act as the nuclei for the enzymatic growth of the silver nanoparticles. The DNA is bound through a (3-glycidyloxypropyl)trimethoxysilane (GOPS) functionalization of the substrate surface. Taking the silicon–oxygen bond length of 0.162 nm into account (see for example [27]), an epoxy group occupies an area of 0.105 nm^2^. The diameter of a B-DNA amounts to approximately 2 nm (see for example [28]), corresponding to an area of a circle of approximately 3.1 nm^2^. In other words, every DNA molecule has the possibility to find a place to bind on the GOPS, because the DNA molecule is more than one order of magnitude larger as compared to the area of one epoxy group from the GOPS.

**Figure 4:**
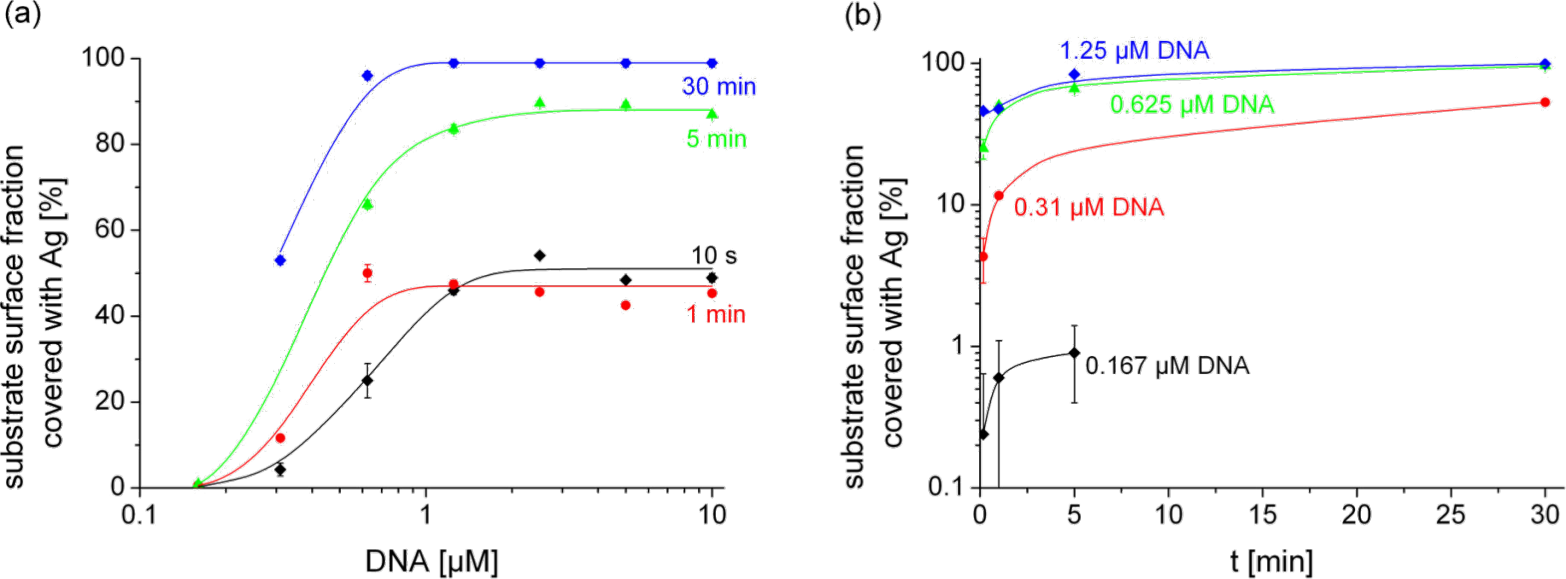
Fraction of the substrate surface covered by silver nanoparticles obtained by grey-tone analysis of SEM images (analysed field size: 12 × 9 µm^2^) in dependence on (a) the initial concentration of DNA with the time of synthesis as a parameter and (b) time of particle growth with the DNA concentration as a parameter. Hidden error bars are too small to be displayed on the chosen axis scale. The curves are only a guide for the eye. Please note the differently scaled ordinates.

In order to enable a deeper insight into the amount of silver bound onto the substrate surface, we estimated the fraction of the substrate surface covered by silver as compared to the total substrate surface, using a grey-tone analysis of the SEM images. For this purpose EGNP samples synthesized for different DNA concentrations and reaction times were investigated. The image size used for the evaluation was 12 × 9 µm^2^ in all cases. The analysis was carried out by using the Java based public-domain software ImageJ [29]. Briefly speaking, after the area for analysis was defined, the 8-bit grey-scale SEM image was converted into a two-tone black and white image by using a threshold function to separate the Ag-EGNP (light grey colour) from the uncovered substrate areas (dark grey to black). The setting of the threshold value was visually controlled in order to minimize the error. The two-tone image was analysed for the area fraction of the Ag-EGNP. Of course the analysed images reveal only a snapshot of all measurements, but they are typical examples from a number of synthesized and measured samples. Figure 4 shows the results of the grey-tone analysis. When interpreting these results one has to take into account that the data represent only the silver distribution in terms of surface area. The curves do not allow conclusions in terms of the amount of deposited silver, because the grey-tone analysis does not consider the height of the silver nanoparticles.

The grey-tone analysis confirms a transition between a low-density array of silver nanoparticles to an almost closed film of nanoparticles for DNA concentrations below 1 µM. The time dependence of the evaluation of the surface coverage affirms a fast increase within the first minute. After 5 min the surface is completely covered by silver nanoparticles for DNA concentrations above 1 µM. But once again, the apparent saturation of the surface coverage only corresponds to the state of a completely closed surface and does not allow a prediction as to whether the silver nanoparticles grow further in the vertical direction.

### Structural investigations of the silver EGNP

**Figure 5:**
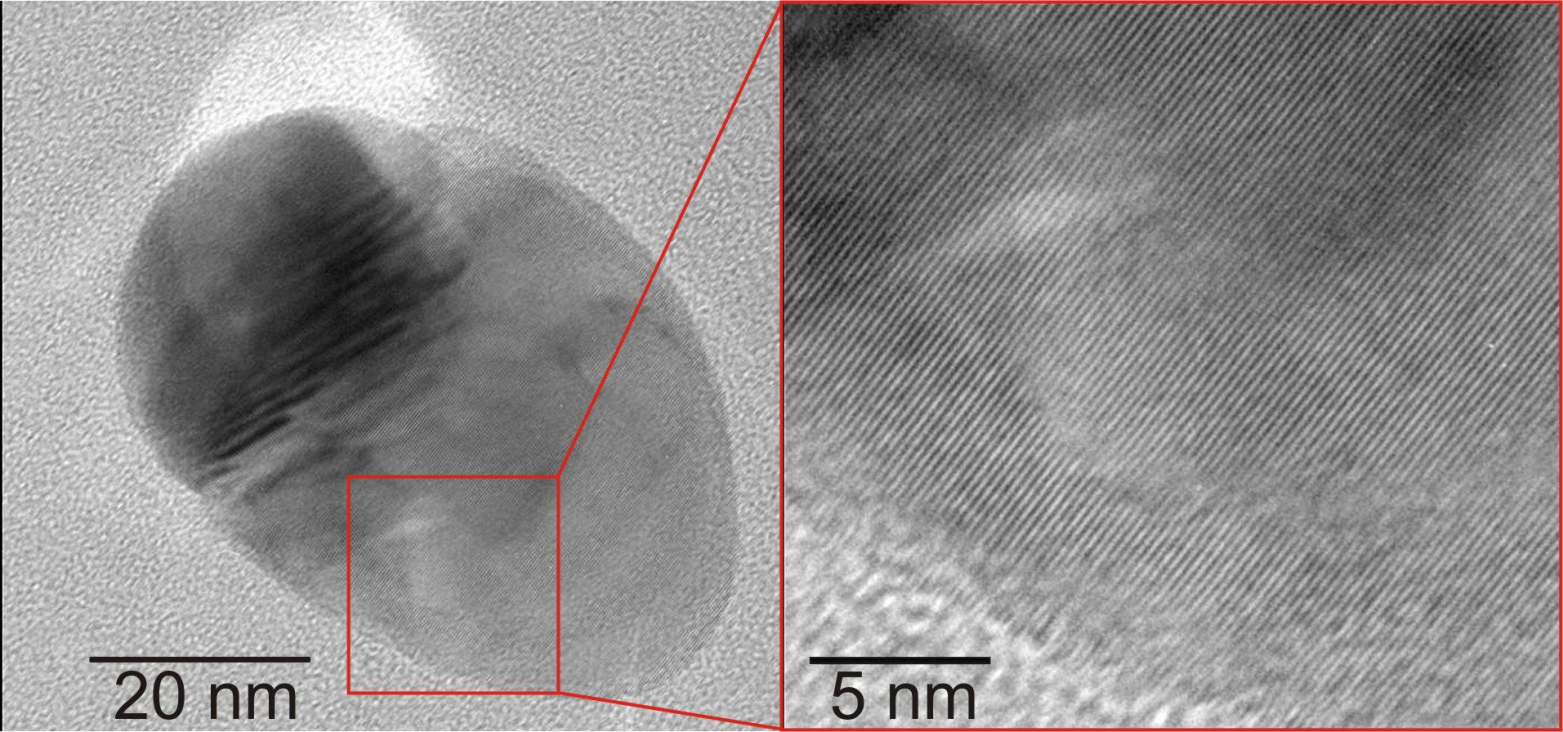
High-resolution TEM image of a silver nanoparticle grown by enzymatic synthesis. The right part shows a detail at higher magnification with clearly observable crystallographic planes of silver.

A more detailed picture of the shape of the single silver nanoparticles was obtained by means of scanning transmission electron microscopy on thinned cross sections. With the help of high-resolution images the crystal structure of the individual silver nanoparticles could be determined. Figure 5 shows a TEM image of a part of a small silver nanoparticle. The right part of the picture displays a high-resolution lattice image. Here the atomic planes of the silver crystal are visible. From the diffraction patterns corresponding to the TEM images the crystal planes can be correlated with silver. That is the silver nanoparticles consist of single-crystalline silver plates. The morphological structure of the silver nanoparticles given by the intertwined plates is not seen in the images, because the TEM cross sections represent only a small part and in the present case only one plate of an entire silver nanoparticle.

### Chemical composition of the silver EGNP

**Figure 6:**
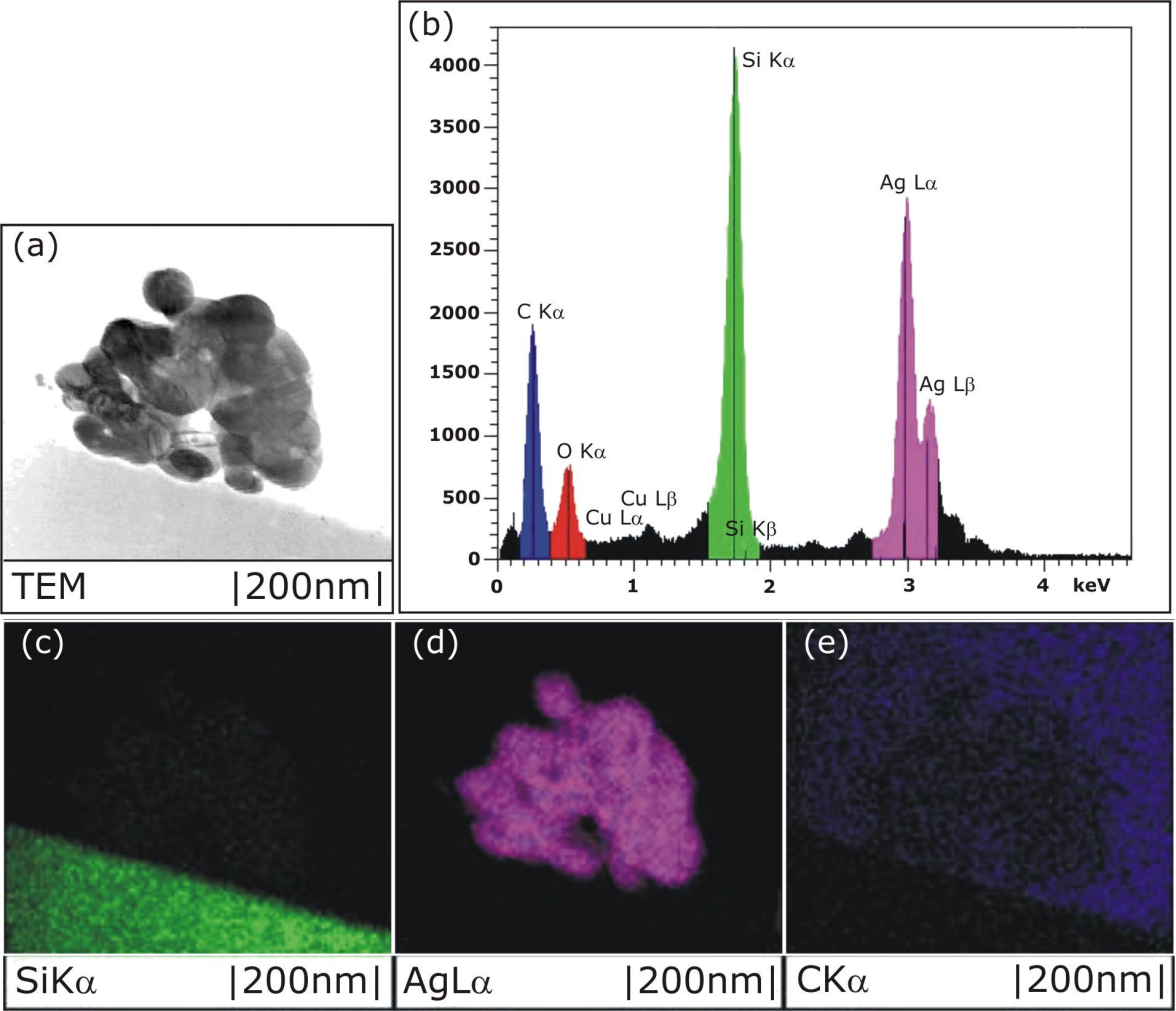
TEM cross section image of a silver nanoparticle on a glass substrate (a), an EDX spectrum recorded across the cross section (b), and mappings of the elements silicon (c), silver (d), or carbon (e), respectively.

In order to determine the chemical composition of an enzymatically grown silver nanoparticle, locally resolved EDX microanalysis was applied during scanning TEM investigations. Figure 6a shows an overview TEM cross-section image of an aggregated silver nanoparticle on a glass substrate. The EDX spectrum of this nanoparticle (Figure 6b) reveals silicon and silver as the main constituents. The carbon peak in the low-energy part of the spectrum results from unavoidable carbon contamination, and the copper signal originates from the TEM grid. Furthermore, an oxygen peak is detected in the low-energy part of the EDX spectrum. The oxygen is due to the SiO_2_ from the glass substrate. Figure 6c–e represent the local distribution of the chemical elements silicon (green), silver (purple), and carbon (blue), respectively. The data are recorded with EDX exactly at the particle shown in Figure 6a. The black colouring in the Figure 6c–e depicts the absence of the dedicated material. In Figure 6c we see that the silicon (green) occurs only in the substrate, taking up the same area as the light grey lower area of Figure 6a, which depicts the glass substrate. Silver appears only in the nanoparticle itself (Figure 6d). The distribution map of carbon (Figure 6e) shows only a small concentration around the nanoparticle and an even lower amount at the cross section of the nanoparticle itself. Thus, the EDX spectrum provides evidence that the nanoparticle consists of silver only.

Beyond the SEM pictures, the TEM cross section images (Figure 5 and Figure 6a) show the real structure and shape of the silver nanoparticles composed of intertwined silver plates. The EDX spectra of these plates show silver Lα and Lβ lines only. Hence, the enzymatically grown silver nanoparticles consist of pure silver. Furthermore, TEM diffraction patterns show the silver plates of the EGNP to be single crystalline.

### RBS analysis for the density of silver in the EGNP

In order to study the integral coverage of the silver nanoparticles on the substrate surface the Rutherford backscattering spectrometry (RBS) technique was applied. RBS is able to deliver significant data about the averaged three-dimensional distribution of chemical elements, which is a clear advantage compared to the aforementioned grey-scale analysis of SEM images or the analysis of AFM data. Firstly, the lateral dimension of the analysed area in RBS measurement is approximately 1 mm^2^ due to the diameter of the ^4^He^+^ ion beam compared to areas on the order of 1 or 100 µm^2^ in the case of AFM or SEM imaging, respectively. Thus, the results derived from RBS data are averaged over areas that are significantly larger, by orders of magnitude, than the silver nanoparticles, in order to avoid errors due to the analysis of small, statistically deviating regions. Secondly, the penetration depth of the 1.4 MeV ^4^He^+^ ions reaches some micrometers due to the relatively low energy loss of the highly energetic ions in solids. Consequently, the whole silver thickness is recorded, if one takes the height of the silver nanoparticles into account, which was derived from the SEM cross-section images (see Figure 3). Thirdly, RBS is able to deliver the absolute number of silver atoms, instead of the percentage of surface that is covered by silver or the maximum height of silver only, as in the case of AFM or SEM data analysis.

**Figure 7:**
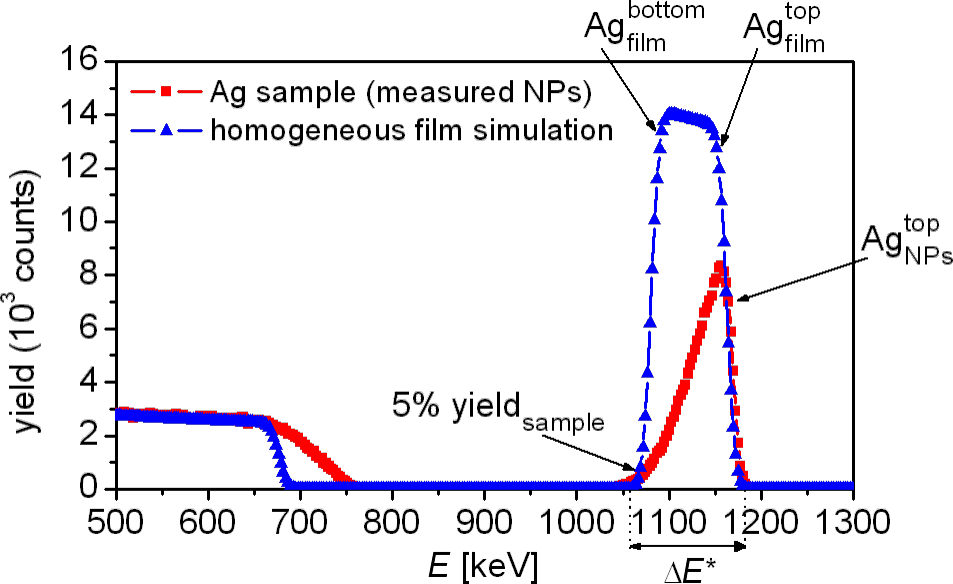
Experimentally recorded RBS spectrum for silver nanoparticles synthesized on a silicon substrate (red squares) together with a RBS simulation of a homogeneous silver film (blue triangles). The left shoulder below 750 keV corresponds with the silicon substrate and the right peaks correspond to the silver thin film or the silver nanoparticles, as appropriate.

In order to explain the RBS measurement, Figure 7 shows a typical RBS spectrum measured for silver nanoparticles deposited on a silicon substrate (red squares) in comparison to a simulated spectrum of a homogeneous silver film (blue triangles). The thickness of the simulated film was adjusted in such a manner that the yield of the simulated film at the low-energy end of the film shoulder is equal to 5% of the maximum yield of the measured film (denoted by 5% yield_sample_). With a maximum yield of the measured film of 8340 counts at 1157 keV the 5%-yield criterion corresponds to 417 counts at 1068 keV. The high-energy part of the spectrum is caused by backscattering of ^4^He^+^ ions by silver atoms at the upper surface of the silver. This is the surface of the simulated homogeneous silver film (denoted by Ag_film_^top^) or the top of the silver nanoparticles (Ag_NPs_^top^), as appropriate. The steep slope of the simulated silver film curve at Ag_film_^bottom^ is due to the interface between the simulated homogeneous silver film and the substrate. Thus, the difference between Ag_film_^top^ and Ag_film_^bottom^ corresponds to the energy loss of the ^4^He^+^ ions within the thickness of the silver film. The width ΔE* for the silver nanoparticles is caused by the energy loss of the ^4^He^+^ ions within the highest silver nanoparticles. For silver nanoparticles of lower height the maximum energy loss is smaller resulting in a triangular peak.

Three main features could be derived from the RBS spectra shown in Figure 7: (1) the integral amount of silver (which is contained in the synthesized silver nanoparticles) by determining the effective area under the silver peak; (2) the percentage of the substrate surface that is covered with the three-dimensional silver nanoparticles independent of their shape, by comparing the yield for the upper surface of the silver nanoparticles (this is the highest peak of the nanoparticles spectrum at approx. 1160 keV) with the high-energy yield for the simulated film curve; and (3) the maximum height *h*_max_ of the silver nanoparticles by comparing the low-energy tail of silver with that of the homogenous silver film.

**Figure 8:**
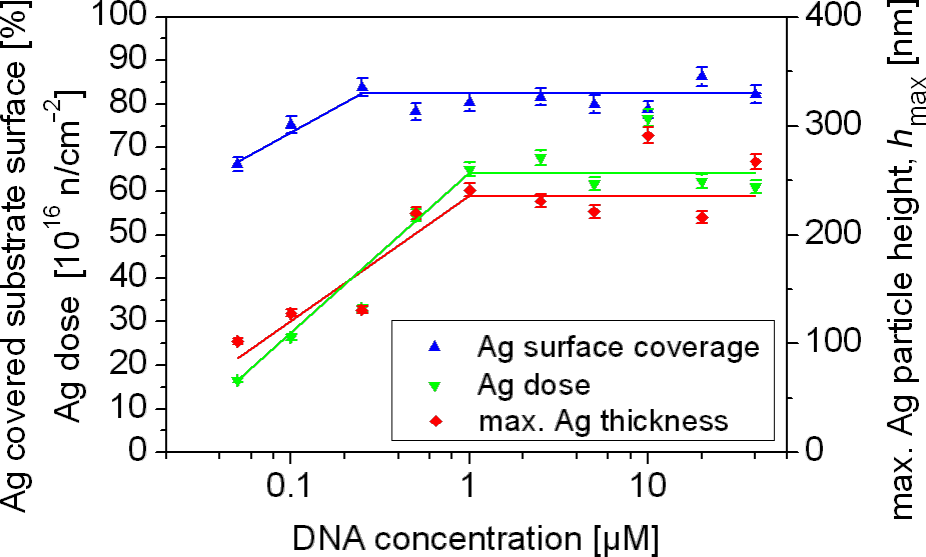
Surface coverage of silver (blue triangles) and absolute number of silver atoms (green circles), both belonging to the left ordinate, as well as the maximum height of the silver nanoparticles assuming the silver bulk density for the nanoparticles (red squares; belonging to right ordinate) of EGNP films on silicon in dependence on the DNA concentration, determined by means of RBS measurements. The reaction time was always 5 min. The lines are only a guide for the eye.

In order to study the surface density and the total particle number (i.e., the dosage) of silver in the nanoparticle films, samples were synthesized with DNA concentrations in the range of 0.05–40 µM. Although we already observed a concentration-independent level of silver coverage for DNA concentrations above 1 µM in the SEM images (Figure 2 and Figure 4), we performed the RBS experiments in dependence on DNA concentration too in order to study the growth of the silver nanoparticles in the *z*-direction normal to the substrate, by determining the maximum height of the nanoparticles and the total amount of silver. The reaction time for the silver nanoparticle growth was set to 5 min for all cases. Figure 8 presents the results of the analysis of the RBS data in terms of the silver-covered surface fraction (blue triangles) in dependence on the DNA concentration. For the investigated reaction time of 5 min the silver surface coverage reaches a stable value of approximately 80–85% for DNA concentrations of 1 µM, which is in accordance with the data derived from the SEM images. Even for high DNA concentrations up to 40 µM the surface coverage does not reach unity, i.e., gaps still exist between the silver nanoparticles. This fact is in correspondence with the SEM images of silver-nanoparticle samples synthesized for 5 min (see the third row of images in Figure 2). The maximum height of the silver nanoparticles was calculated from the RBS spectra. By doing so a homogenous silver film with the same silver dose as the measured sample was simulated such that the energy value at 50% of the maximum yield of the simulated film was equal to the energy value at 5% of the maximum yield of the measured sample, i.e., *E*(50% yield_film_) = *E*(5% yield_sample_) = E* (see Figure 7 for explanation). The thickness of this simulated film was taken as the maximum height of the silver nanoparticles. The calculated thickness (red squares) increases strongly for DNA concentrations below 1 µM, reaching a value of about 250 nm after 5 min, independent of the DNA concentration. The estimates from the RBS data as well as from the aforementioned grey-tone analysis of the SEM images indicate a saturation of the silver-covered surface, but they do not allow a statement concerning the total amount of synthesized silver. The maximum height of the silver nanoparticles derived from the RBS data provides evidence for saturation with respect to the silver nanoparticle growth. To confirm this, the RBS data were analysed with respect to the integral silver dose, i.e., the total number of silver atoms (green data in Figure 8). For DNA concentrations larger than 1 µM the behaviour appears to be independent of the DNA concentration. However, it should be mentioned, that all the RBS data were obtained for samples that were synthesized over a constant time of 5 min.

A number of samples were measured again after two months and we found a reproducibility of the RBS spectra within about 2 % indicating stable silver structures.

### SERS detection of riboflavin by using silver EGNP arrays

**Figure 9:**
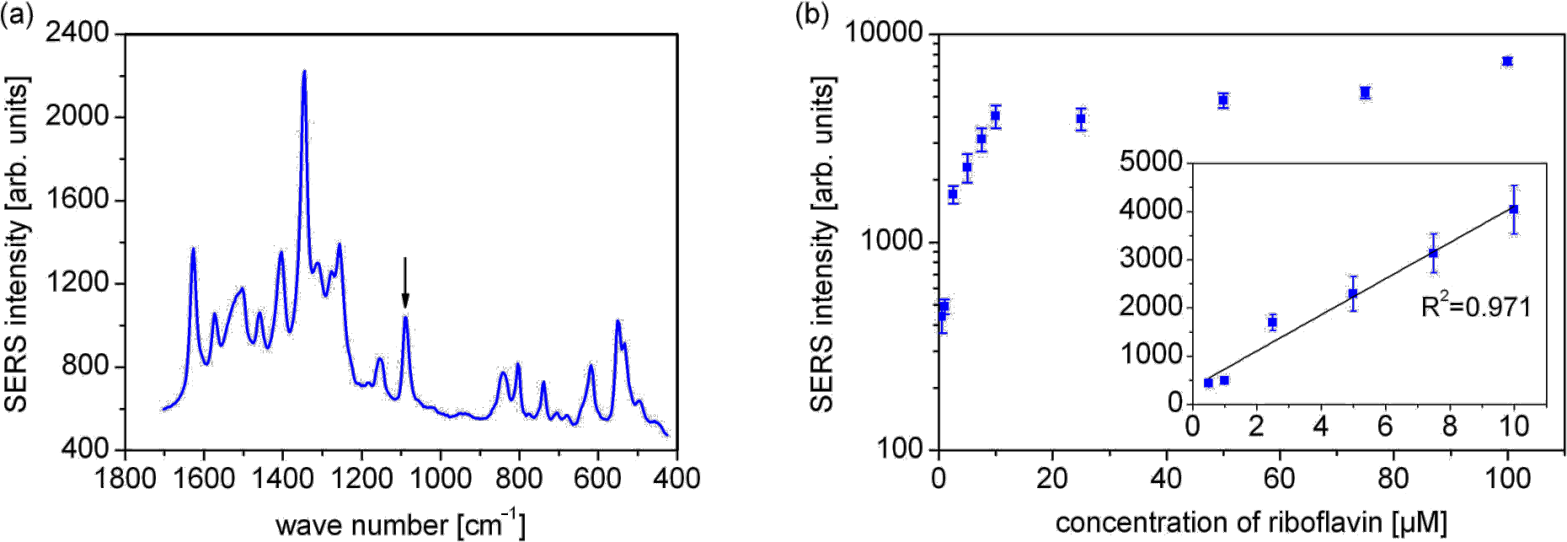
(a) A typical SERS spectrum of riboflavin (5 µM) on enzymatically generated silver nanoparticles measured for an excitation wavelength of 532 nm and (b) the dependence of the integrated SERS intensity at 1087 cm^−1^ (flagged with the arrow in part (a)) on the riboflavin concentration. The inset shows the strongly increasing SERS intensity for low riboflavin concentrations on a linear scale.

Enzymatically generated silver nanoparticles are suitable as surface-enhanced Raman scattering (SERS) substrates. Recently Strelau et al. reported the correlation between electrical conductivity and SERS activity [7]. For a fast, specific, and sensitive detection of molecules at low concentration, EGNP were applied for qualitative as well as quantitative SERS measurements. In order to demonstrate the SERS activity of EGNP arrays, SERS measurements of the vitamin riboflavin were performed. The silver nanoparticles were generated as described above for a DNA concentration of 10 µM and a reaction time of 5 min for the silver deposition. Furthermore, the substrates were incubated for one hour with 100 µl of an aqueous solution of riboflavin at different concentrations between 0.5 to 100 µM. Afterwards the substrates were dried under a stream of nitrogen. The SERS measurements were performed at an excitation wavelength of 532 nm. Figure 9a depicts a SERS spectrum of riboflavin (5 µM). In order to show the signal evolution in dependence on the concentration, the integrated Raman intensity of the mode at 1087 cm^−1^ is plotted as a function of the riboflavin concentration. A linear correlation occurs for the low concentration range up to 10 µM (inset in Figure 9b) whereas high concentration levels feature only a weak concentration dependency towards saturation. The SERS signal saturation may be explained by an oversaturation of the binding sites on the surface. At high concentrations, the molecules are located in several layers on the surface, whereas only molecules in the first layer next to the silver nanoparticles undergo the most efficient signal amplification. The simplest approach for quantitative investigations may be to carry out SERS measurements of solutions only in such concentration ranges, in which all binding sites of the SERS substrate are saturated and where no oversaturation can occur. Thus, EGNP arrays can be applied for quantitative SERS measurements at low concentrations.

## Conclusion

The enzymatically grown silver nanoparticles were shown to consist of pure silver. This is an important result for further applications of the silver nanoparticles for bio-analytics since the nanoparticles are free from contaminations. The building blocks of the individual nanoparticles are single-crystalline silver plates, which are intertwined to form nanoflower or desert-rose-like nanoparticles up to a size of some hundred nanometers depending on the conditions of the synthesis. Within the applied preparation conditions DNA concentrations of 1 µM and reaction times below 5 min are sufficient to obtain films of densely packed silver nanoparticles.

The enzyme-induced growth of metal nanoparticles may lead to building blocks for spectroscopic applications that give stable signals that do not fade or bleach. One field of application of the silver nanoparticles, grown in an enzyme-induced process, is in the preparation of SERS active substrates, for example. An advantage of the illustrated method to synthesize the silver nanoparticles on a surface by enzymatic growth, is the simple preparation from a wet solution instead of by using vacuum deposition techniques, such as evaporation to fabricate rough metal films, or complicated top-down methods of micro- or nanopatterning of previously deposited thin metal films. This simplicity can help to extend the range of applications of enzymes in bioanalytics due to the generation of insoluble, stable products. Successful concentration-dependent SERS measurements on the vitamin riboflavin have been shown on such SERS-active substrates.

## Experimental

### Substrate preparation

The silver EGNP were synthesized on glass or silicon substrates. The silicon substrates, which were used for the RBS investigations in order to avoid any electrical charging during analysis, were covered by a natural oxide layer. The coarse cleaning of the substrates was performed in an ultrasonic bath in acetone, ethanol, and water (for 10 min each) followed by drying under nitrogen. Next, the surface was activated in an oxygen plasma etching step. In order to bind linker molecules (amino modified DNA) onto the surface, the substrates were chemically modified with 10 mM (3-glycidyloxypropyl)trimethoxysilane (GOPS) in dried toluene for 3 h at 70 °C, followed by three washing steps in toluene. As a result, the surface was saturated with epoxy groups. Afterwards, linker molecules (here: DNA with a 5-end amino and a 3-end biotin modification) were deposited on the surface as 4 nl droplets in 5× phosphate-buffered saline (PBS) by using a Nano-Plotter NP 2.0 (GeSiM mbH, Großerkmannsdorf, Germany). The droplets dried under normal atmosphere within 10 min. The linker binds covalently via the amino modification towards the epoxy modified surface, assisted by an UV linking process [26]. Due to the interaction of biotin and streptavidin, a streptavidin horseradish peroxidase conjugate complex is bound via the linker molecule to the surface. In order to remove the remaining unbound peroxidase complex, the samples were washed six times in PBST buffer for 2 min each after 1 h incubation of the surface with the peroxidase complex. Finally, the substrates were rinsed with distilled water. The concentration of the linker was varied throughout the experiments, whereas the concentration of the peroxidase complex was kept constant at 10^−3^ mg/ml. All preparation steps were performed at room temperature.

#### Synthesis of the enzymatically grown nanoparticles (EGNP)

The silver nanoparticles were prepared subsequently to the peroxidase coupling. Silver nanoparticles were generated by using the silver enhancement kit (EnzMet^TM^) from Nanoprobes (Nanoprobes Inc., Yaphank, NY, USA). Here, the horseradish peroxidase catalyzed the silver reduction. Thus, the addition of the EnzMet^TM^ enhancement kit immediately led to the formation of silver nanoparticles by the enzymatic activity. Within these studies, different growth times were used for the silver nanoparticles. In order to stop the silver deposition process, the substrates were rinsed with water. Finally, the substrates were dried under nitrogen. The preparation was previously described in other publications [6–7].

#### Scanning and transmission electron microscopy

The scanning electron microscopy (SEM) images were recorded by means of a JEOL JSM-6300F using a 5 kV electron beam. Transmission electron microscopy (TEM) cross-section images were recorded with a Philips CM 20 FEG. The TEM with a field emission gun was operated at an acceleration voltage of 200 kV. During the TEM analysis local energy dispersive X-ray (EDX) spectra were taken at different points in the TEM lamellas. For the TEM investigations thin cross sections were prepared by means of the focused-ion-beam etching technique. For the cross-section images, samples were prepared on glass substrates with DNA concentrations of 10 µM. The silver reaction time was 5 min.

#### Rutherford backscattering spectrometry (RBS)

RBS is based on the scattering of high-energy ions in solids. For this purpose a beam of high energy ^4^He^+^ ions is directed onto the target, i.e., the sample of interest, at a well-controlled angle of incidence. The ^4^He^+^ ions are scattered by the nuclei within the target. The recoil energy of the ^4^He^+^ ions is a unique function of scattering angle, initial kinetic energy, and target nucleus mass. The energy and quantity of backscattered ions are detected in a certain solid angle in the backward direction almost antiparallel to the incident beam. The determination of the atomic mass of the scattering centres, and hence the chemical element, is possible, since the energy of the elastically scattered ions increases with the mass of the scattering centres. Depth resolution can be achieved due to the additional energy loss of the ions based on inelastic scattering. The depth resolution depends on the energy resolution of the detector (in our case FWHM = 12 keV) and on the material being measured, and is typically on the order of several nanometers for silver particles. For measurement and analysis of the measured data the software RUBSODY was used [30]. The ion energy of the ^4^He^+^ beam was 1.4 MeV; the angle between the incident beam and the detected backscattered ions was 168°. The area of measurement was approximately 1 mm in diameter, corresponding to the spot of the incident ^4^He^+^ ion beam.

#### Surface-enhanced Raman spectroscopy (SERS)

The SERS spectra were recorded with a microRaman setup (HR LabRam invers, Jobin-Yvon–Horiba) by using a frequency-doubled Nd:YAG laser (λ = 532.11 nm) as excitation source. The spectrometer is equipped with an entrance slit of 100 µm, a focal length of 800 mm and a 300 lines/mm grating. SERS measurements were carried out by focusing the laser light onto the samples (approx. 0.7 µm focus diameter) with a Leica PLFluoar H100 objective with a laser power of 25 µW and an integration time of 1 s. A total of 250 spectra per sample were recorded in the line-scanning mode (five line scans with 50 spectra per line). A CCD camera operating at 220 K was used to detect the Raman scattered light.
